# Characterization of Nitric Oxide-Inducing Lipid A Derived from *Mesorhizobium loti* Lipopolysaccharide

**DOI:** 10.1264/jsme2.ME12103

**Published:** 2012-10-10

**Authors:** Masahito Hashimoto, Youhei Tanishita, Yasuo Suda, Ei-ichi Murakami, Maki Nagata, Ken-ichi Kucho, Mikiko Abe, Toshiki Uchiumi

**Affiliations:** 1 Department of Chemistry, Biotechnology, and Chemical Engineering, Kagoshima University, Korimoto 1–21–40, Kagoshima 890–0065, Japan; 2 Department of Applied Biology and Chemistry, Tokyo University of Agriculture, Sakuragaoka 1–1–1, Setagaya, Tokyo 156–8502, Japan; 3 Department of Environmental Sciences, Faculty of Agriculture, Saga University, Honjyo-machi 1, Saga 840–8502, Japan; 4 Department of Chemistry and Bioscience, Kagoshima University, Korimoto 1–21–35, Kagoshima 890–0065, Japan

**Keywords:** lipid A, lipopolysaccharide, MS, NMR, nitric oxide

## Abstract

*Mesorhizobium loti* is a member of the rhizobia and forms nitrogen-fixing symbioses with several *Lotus* species. Recently, it was reported that *M. loti* bacterial cells and their lipopolysaccharide (LPS) preparations transiently induced nitric oxide (NO) production in the roots of *L. japonicus*. We subsequently found that polysaccharides and the lipid A moiety were responsible for this NO induction. In this study, we elucidated the chemical structure of *M. loti* lipid A and characterized its NO-inducing activity in response to structural modifications. *M. loti* LPS were partially hydrolyzed with hydrazine or aqueous hydrofluoric acid to obtain O-deacylated or dephosphorylated LPS, respectively. The untreated and treated LPS fractions were subjected to weak acid hydrolysis to obtain lipid A fractions. The chemical structure of *M. loti* lipid A was elucidated by chemical composition analysis, MALDI-TOF-MS, and NMR spectra to be P-4-β-GlcNN(1-6)α-GlcNN(1-1)α-GalA, in which positions 2 and 3 of β-GlcNN are substituted for 3-acyloxy-fatty amides, and positions 2 and 3 of α-GlcNN are substituted for 3OH-fatty amides. The partial hydrolysis of lipid A appeared to reduce its NO-inducing activity. These results suggest that *L. japonicus* root cells recognize the lipid A structure as a means of controlling NO production.

Nitric oxide (NO) is a gaseous signaling molecule that exerts a wide range of regulatory functions in many organisms from plants to mammals. In plants, NO is known to be implicated in the defensive responses to pathogen invasion (18). Leaf and cell suspensions derived from *Arabidopsis*, soybean, or tobacco rapidly accumulated NO after treatment with bacterial, fungal, or viral organisms, elicitors, or components. In combination with other reactive oxygen species, increased NO expression activates hypersensitive reactions in plants, resulting in the formation of necrotic lesions at infected sites. NO is also involved in the expression of defense-related genes including phenylalanine ammonia lyase, chalcone synthase, and pathogenesis-related protein PR-1, which induce local and systemic defense mechanisms (11).

During the last decade, many reports demonstrated that NO production is involved in symbiotic plant–microbe interactions, particularly in legumes and nitrogen-fixing rhizobium (12). Previously, Shimoda *et al.* (22) characterized a *Lotus japonicus* class 1 non-symbiotic hemoglobin, LjHb1, which is considered to be able to control NO levels in plants. High LjHb1 expression was observed in mature root nodules, and *LjHb1* expression was induced under stress conditions, including hypoxia, cold, and NO exposure. *LjHb1* expression was also induced by the inoculation of *Mesorhizobium loti* MAFF303099, a symbiont of *L. japonicus*. Furthermore, Nagata *et al.* (15) observed transient (up to 4 h) NO production, and *LjHb1* expression was also observed in roots inoculated with *M. loti*. Conversely, the plant pathogens *Ralstonia solanacearum* MAFF730135 and *Pseudomonas syringae* MAFF730032 stimulated continuous NO production in the roots of *L. japonicus* for at least 24 h, without inducing *LjHb1* expression. Neither *LjHb1* expression nor NO production was observed in the roots of *L. japonicus* in response to inoculation with the non-symbiotic *Sinorhizobium meliloti*. These observations suggest that transient NO production and *LjHb1* expression participate in the recognition of symbiotic rhizobia by legumes.

Recently, Nagata *et al.* (14) showed that the crude lipopolysaccharide (LPS) fraction of *M. loti* induced NO production in *L. japonicus* roots. LPS are macroamphiphilic molecules that are distributed on the outer membranes of most Gram-negative bacteria and act as pathogenic factors in mammals and plants (23). Since LPS are located on the surfaces of bacterial cells, it is reasonable that they are one of the first molecules recognized by plant cells. LPS are generally composed of a hydrophilic polysaccharide component, which consists of an O-antigenic polysaccharide and a core oligosaccharide, and a membrane-anchoring lipid A moiety, which is covalently attached to 2-keto-3-deoxyoctulosonic acid (KDO), the reducing end sugar of the polysaccharide component. Murakami *et al.* (13) further demonstrated that LPS and their acid hydrolysate, polysaccharide, and lipid A fractions participate in the induction of NO production in the roots of *L. japonicus*; *i.e.*, NO production was detected in *L. japonicus* roots 4 h after their treatment with any of the above-mentioned fractions. NO production was also detectable in the roots 24 h after treatment with LPS or lipid A, but not after treatment with the polysaccharide fraction. These observations suggest that LPS exerts dual functions; *i.e.*, the lipid A moiety acts as a continuous NO inducer, and the polysaccharide component acts as a transient NO stimulator; however, the structural requirements for each role are poorly understood. In this study, we elucidated the chemical structure of *M. loti* lipid A and characterized its NO-inducing activity in response to structural modifications.

## Materials and Methods

### Preparation of the LPS fraction and lipid A

The LPS fraction from *Mesorhizobium loti* MAFF303099 was prepared using a previously described method (13). Briefly, the bacterial cells were subjected to phenol–hot water extraction to obtain aqueous and phenolic phases. The extracts of the phenolic phase and aqueous phase were digested with DNase and RNase, followed by proteinase K, and purified by hydrophobic interaction chromatography on Octyl Sepharose 4FF or ultracentrifugation, respectively.

Modified LPS fractions were obtained by 47% aqueous hydrofluoric acid (HF) or anhydrous hydrazine (HZ) treatment. A sample of the crude LPS fraction (10 mg) was hydrolyzed with 50 μL HF at 4°C for 24 h and lyophilized over NaOH. Another sample of the crude LPS fraction (10 mg) was hydrolyzed with 1 mL HZ at 37°C for 18 h and precipitated with 10 mL cold acetone. An *Escherichia coli* O55:B5 LPS preparation was purchased from Sigma-Aldrich (St. Louis, MO, USA).

The LPS fraction was hydrolyzed with 0.6% acetic acid at 105°C for 2.5 h and centrifuged at 5,700×*g* (F0850 rotor; Beckman Coulter, Brea, CA, USA) to yield crude lipid A as a precipitate. The precipitate was separated by Silica Gel 60 column chromatography (Kanto Chemical, Tokyo, Japan) using a solvent system composed of chloroform–methanol–water (65:25:4 [v/v/v]) to obtain the lipid A fraction.

### Analytical procedures

The sugar constituents of each fraction were analyzed using a 4-aminobenzoic acid ethyl ester (ABEE) method (27). Samples of each fraction were hydrolyzed with 4 M trifluoric acid (TFA) at 100°C for 6 h and derivatized with ABEE using the standard method. The ABEE-labeled sugars were analyzed by HPLC.

The fatty acid constituents of each fraction were analyzed according to the methyl ester method (10). The samples were methanolyzed with 5% hydrogen chloride-methanol at 100°C for 3 h or 18 h. The fatty acid methyl esters in the samples were analyzed by gas chromatography (GC) or GC-mass spectroscopy (GC-MS).

Analytical thin layer chromatography (TLC) was performed on a TLC plate (No. 5715; Merck, Darmstadt, Germany) using a solvent system consisting of chloroform-methanol-water (65:25:4 [v/v/v]), and the resultant spots were visualized with anisaldehyde-sulfuric acid reagent.

### Nuclear magnetic resonance (NMR) and mass spectrometry (MS)

^1^H, ^13^C, and ^31^P NMR spectra were obtained at 600, 151, and 243 MHz, respectively, on a JMN-ECA600 spectrometer (JEOL, Tokyo, Japan) equipped with an HX5 indirect detection gradient probe. The spectra were obtained at 327K in dimethyl sulfoxide (DMSO)-d_6_/CDCl_3_ (1:1 [v/v]). The chemical shifts are expressed as δ values using DMSO (δ2.49) as an internal standard for ^1^H NMR spectra, and DMSO (δ39.7) was also used as an internal standard for the ^13^C NMR spectra. For the ^31^P NMR spectra, 85% phosphoric acid was used as an external standard (δ0). The signals were assigned using correlation spectroscopy (COSY), totally correlated spectroscopy (TOCSY), nuclear Overhauser effect spectroscopy (NOESY), hetero-nuclear multiple quantum coherence (HMQC), ^1^H-^13^C heteronuclear multiple-bond connectivity (HMBC), and ^1^H-^31^P HMBC spectra. The coupling constants were determined by one-dimensional ^1^H NMR in combination with J-resolved spectra.

Matrix-assisted laser desorption/ionization time-of-flight mass spectrometry (MALDI-TOF-MS) spectra were obtained using a Voyager DE instrument (Applied Biosystems, SCIEX, Toronto, Canada). The samples were dissolved in dichloromethane-methanol (3:1 [v/v]) combined with 2,5-dihydroxybenzoic acid as a matrix and then placed on sample plates. The spectra were obtained in negative ion reflector mode.

### Detection of NO production

NO production in the roots of *L. japonicus* was measured using a 4-amino-5-methylamino-2′,7′-difluorescein diacetate (DAF-FM DA) method, as described previously (13). Briefly, each sample was suspended in water and applied to 14-day-old plants. After incubation for the indicated time, DAF-FM DA was added to the roots. After 30 min incubation, fluorescent images were captured using a Leica DMLB microscope equipped with a Leica DC2000 digital camera. The data are shown as the mean ± SE values obtained from three independent experiments. Statistical significance was analyzed using Welch’s *t* test.

## Results

### Preparation of *M. loti* lipid A and its derivatives

**Fig. 1:**
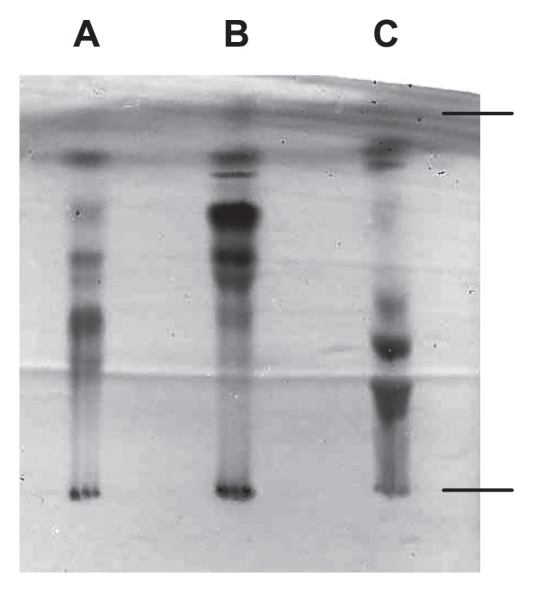
TLC profiles of the hydrophobic precipitates obtained by weak acid hydrolysis. A, untreated LPS; B, HF-treated LPS; C, HZ-treated LPS. TLC was performed using a solvent system consisting of CH_3_Cl-CH_3_OH-H_2_O (65:25:4 [v/v/v]) and visualized using anisaldehyde-sulfuric acid reagent.

**Fig. 2:**
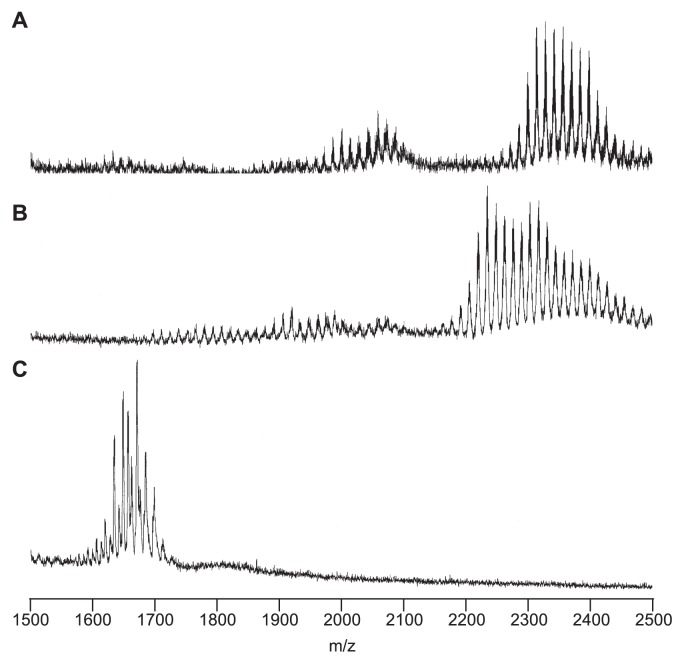
MALDI-TOF-MS spectrum of the hydrophobic precipitates obtained by weak acid hydrolysis. A, untreated LPS; B, HF-treated LPS; C, HZ-treated LPS. Spectra were obtained with a Voyager DE instrument in negative ion refractor mode.

LPS was prepared from *M. loti* MAFF303099 by phenolhot water extraction followed by hydrophobic interaction chromatography, as described previously (13). Since both the aqueous and phenolic extracts contained LPS and their shorter O-antigenic homologue lipooligosaccharides, both preparations were used as LPS fractions for the subsequent separation experiments. The LPS fraction was then subjected to weak acid hydrolysis, which cleaves the acid labile KDO bond to afford a hydrophobic precipitate and hydrophilic polysaccharide components. The thin-layer chromatography (TLC) profile of the LPS hydrolysate precipitate demonstrated the existence of several components (Fig. 1). The MALDI-TOF-MS spectrum of the precipitate, which was obtained in negative mode, displayed multiple ion peaks in the m/z ranges 1900–2200 and 2250–2500 (Fig. 2A). These peaks presumably represented lipid A molecules, as described in a previous report (6). The neighboring peaks in each group displayed differences in their m/z values of 14, which corresponds to methylene (CH_2_), indicating the heterogeneous substitution of fatty acids. The difference in the mean m/z values of the two groups was about 300, which probably indicated the loss of a fatty acid moiety. In addition, a sample of the lipid A fraction was purified by silica gel column chromatography to remove free lipids and polar constituents. This fraction was designated the untreated (UT) lipid A fraction.

Lipid A commonly consists of O-acylated fatty acids, N-acylated fatty acids, and phosphate groups (23). To reduce the complexity of lipid A, various structural modifications were induced. For example, the LPS fraction was treated with aqueous hydrofluoric acid (HF) or anhydrous hydrazine (HZ) to liberate the phosphate and O-acylated fatty acids, respectively, before being subjected to weak acid hydrolysis. The TLC profiles of the precipitates indicated that the structure of lipid A had undergone several modifications (Fig. 1B, C). In the MALDI-TOF-MS spectrum of the hydrophobic precipitate obtained from the hydrolysate of HF-treated LPS, an ion peak decrease of about m/z 80 compared to the UT lipid A peaks was observed, which confirmed the occurrence of dephosphorylation (Fig. 2B). In the MALDI-TOF-MS spectrum of the HZ-treated LPS hydrolysate, the m/z difference between the ion peaks of the UT and HZ-treated lipid A fractions was about 700, which corresponds to the liberation of fatty acids (Fig. 2C). The HF- and HZ-treated lipid A fractions were also purified by silica gel column chromatography.

### Elucidation of the sugar backbone of *M. loti* lipid A

**Fig. 3:**
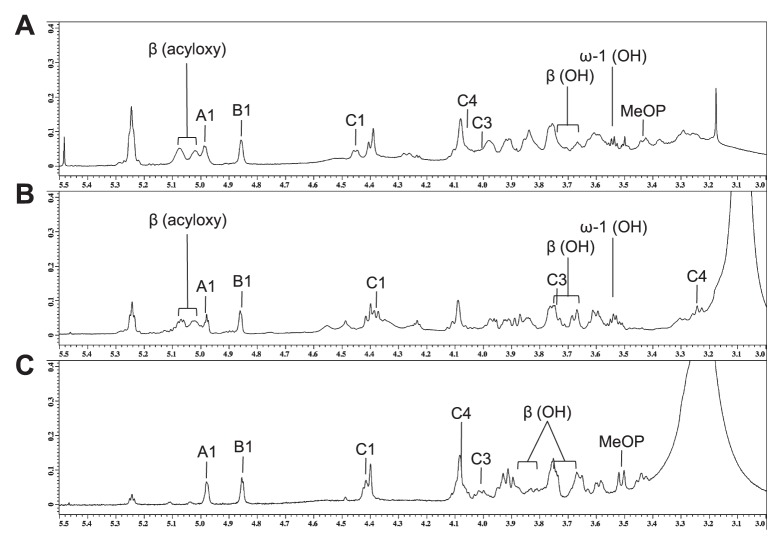
Sections of the one-dimensional ^1^H NMR spectra of lipid A fractions. A, untreated lipid A fraction; B, HZ-treated lipid A fraction; C, HF-treated lipid A fraction. Spectra were obtained at 327K in DMSO-d_6_/CDCl_3_ (1:1 [v/v]).

**Fig. 4:**
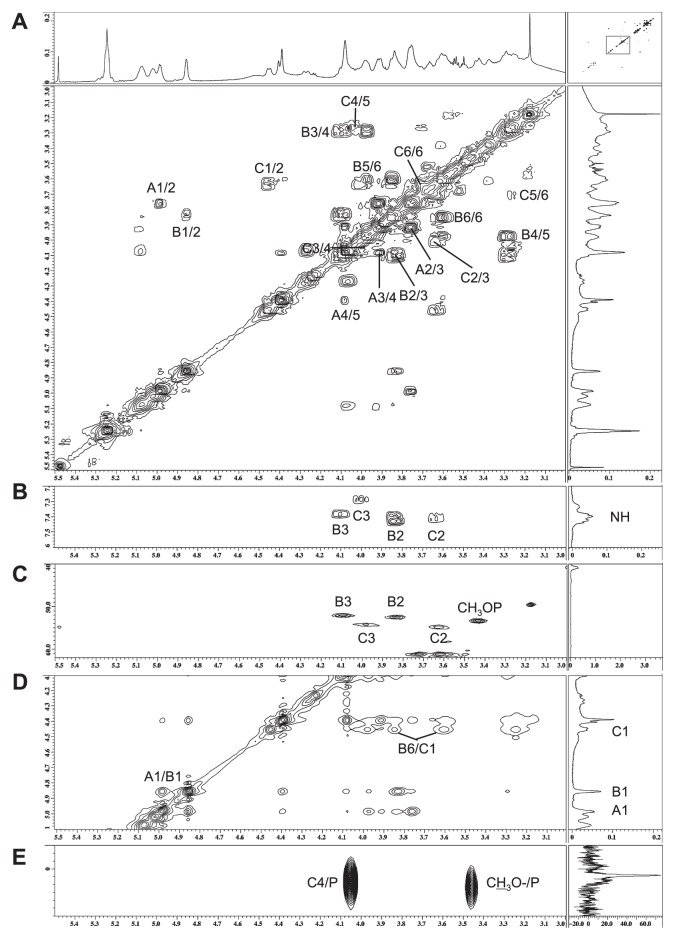
Part of the 2-D ^1^H NMR spectrum of the untreated lipid A fraction. A and B, COSY; C, HMQC; D, NOESY; E, ^1^H-^31^P HMBC spectra. Spectra were obtained at 327K in DMSO-d_6_/CDCl_3_ (1:1 [v/v]).

Sugar analysis of *M. loti* lipid A and its derivatives showed that they contained galacturonic acid (GalA) and an unidentified amino sugar. Thus, their backbone sugars were mainly analyzed by NMR spectroscopy. Sections of their one-dimensional NMR spectra are shown in Fig. 3. The ^1^H and ^13^C NMR spectra of lipid A were assigned using COSY, TOCSY, NOESY, J-resolved, HMQC, ^1^H-^13^C HMBC, and ^1^H-^31^P HMBC, and the resultant data are summarized in Table 1. Three sets of sugar signals (A, B, and C) were observed in the COSY spectrum of UT lipid A (Fig. 4A). Spin system A consisted of five protons and carbons and one carbonyl carbon, which is indicative of GalA. An α-linkage was assigned to the anomeric proton at δ4.98 due to its relatively large ^1^*J*_C,H_ value (176.3 Hz) and relatively small ^3^*J*_H,H_ value (3.5 Hz) (1, 25). Thus, sugar A was determined to be α-GalA. Spin systems B and C both consisted of 7 protons and 6 carbons (Fig. 4A). The four carbons at B2, B3, C2, and C3 displayed signals at δ52–55, indicating that both sugars had diamino-dideoxy hexose structures (Fig. 4C). These NMR data suggested that sugars B and C were 2,3-diamino-2,3-dideoxyglucose (GlcNN) (6). An α-linkage was assigned to the anomeric proton at δ4.86 in the ring of B due to its relatively large ^1^*J*_C,H_ value (177.1 Hz) and its relatively small ^3^*J*_H,H_ value (3.8 Hz), whereas the relatively small ^1^*J*_C,H_ value (161.6 Hz) and relatively large ^3^*J*_H,H_ value (8.2 Hz) of the anomeric proton at δ4.46 in the ring of C was indicative of a β-linkage. Thus, sugar B was determined to be α-GlcNN, and sugar C was considered to be β-GlcNN.

The inter-residual NOESY coupling from proton A1 to B1 confirmed the occurrence of glycosylation between position 1 of α-GalA and position 1 of α-GlcNN, and that from B6 to C1 revealed the presence of glycosylation between β-GlcNN and position 6 of α-GlcNN (Fig. 4D). The heteronuclear correlation detected by ^1^H-^31^P HMBC between the C4 proton and the phosphate group at δ1.2 was suggestive of a phosphate substitution at position 4 of β-GlcNN (Fig. 4E). Another phosphate group was observed at δ2.0 and was found to be correlated with the doublet proton (J=10.5) at δ3.46 by ^1^H-^31^P HMBC (Fig. 4E). A heteronuclear correlation between the proton and the carbon at δ53.4 was also observed (Fig. 4C). The proton did not display any further homonuclear correlations on COSY or TOCSY examinations, indicating that the above findings represented the methyl substitution of the phosphate group, as described previously (17). Since the area of the phosphate peak at δ2.0 was smaller than that at δ1.2, it was considered that the phosphate group had been partially substituted for a methyl group. These results indicated that lipid A has the following sugar backbone: (MeO)_x_-P-4-β-GlcNN(1-6)α-GlcNN(1-1)α-GalA, where x might be less than one-third. The lipid A derivatives were also determined to have the same backbone structure, except for the absence of a phosphate group in the HF-treated lipid A, in which the protons at C4 and C3 were shifted upfield (Fig. 3, Table 1).

### Elucidation of the fatty acid substitutions of *M. loti* lipid A

The fatty acids of lipid A were analyzed by methyl ester methods using GC-MS. The assignment was performed according to the methods of previous reports (3, 5). The fatty acids of the HZ-treated lipid A fraction mainly consisted of 3-hydroxy (3OH) ^i^13:0 and 3OH ^i^20:0, which are amide-linked fatty acids, and those of UT lipid A additionally contained ^i^17:0, 18:1, 18:0, 20:0, 22:0, and 27OH 28:0, which are ester-linked fatty acids. In the COSY spectra (Fig. 4B), four NH protons were found to be correlated to B2, B3, C2, and C3, indicating that all of the amino groups of GlcNN were N-acylated.

**Fig. 5:**
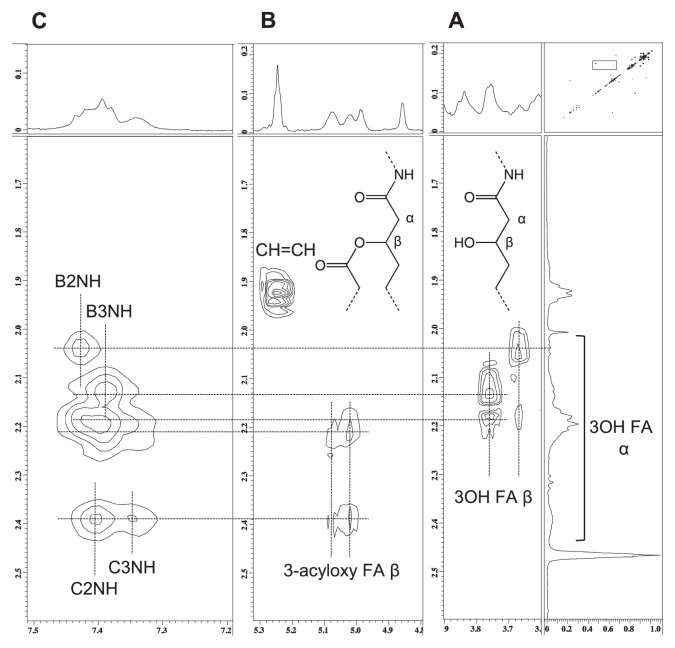
Part of the 2-D ^1^H NMR spectrum of the untreated lipid A fraction. A and B, COSY; C, NOESY spectra. Spectra were obtained at 327K in DMSO-d_6_/CDCl_3_ (1:1 [v/v]).

In the COSY spectra of UT lipid A, the four protons at δ3.65, 3.76, 5.02, and 5.08 were assigned to the β positions of 3OH fatty acids (Fig. 5A, B). The protons at δ5.02 and 5.08 were considered to be derived from 3-acyoxy-fatty acids because of their downfield shifts, whereas the other two were considered to belong to fatty acids with free 3OH groups. The former group was correlated with the α positions of the fatty acids at δ2.2 and 2.4, whereas the latter group was coupled with those at δ2.0–2.2. In the NOESY spectra, the former group was correlated with the NH protons of C2 and C3, and the latter group was linked to those of B2 and B3 (Fig. 5C). These results suggest that the 2 and 3 positions of β-GlcNN were substituted for 3-acyloxy-fatty amides, and those of α-GlcNN were substituted for 3OH-fatty amide residues; *i.e.*, UT lipid A contains two O-linked fatty acids and four N-linked fatty acids. Furthermore, the ω-1 position of the 27OH 28:0 fatty acid was also found to take a free OH form (Fig. 3A) (8), indicating the absence of 3-hydroxybutylate substitution.

The HF-treated lipid A displayed similar results (Fig. 3B). In contrast, the HZ-treated lipid A did not display any β protons belonging to 3-acyloxy-fatty amide substituents or ω-1 protons belonging to 27OH 28:0 fatty acids (Fig. 3C), indicating that it contained four N-linked fatty acids and no O-linked fatty acids.

### Effects of lipid A structural modifications on NO induction

**Fig. 6:**
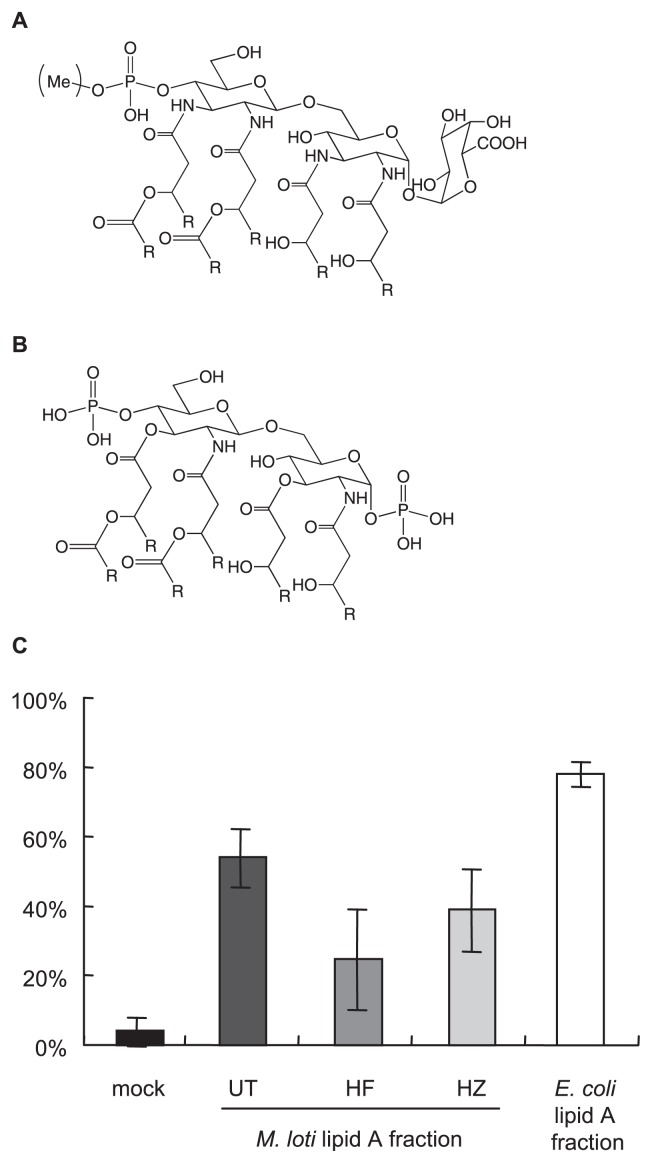
The proportion of NO-producing *L. japonicus* roots 4 h after lipid A fraction (1 mg mL^–1^) treatment. Data are shown as the mean ± SE values obtained from three independent experiments. UT: untreated, HF: HF-treated, HZ: HZ-treated.

The structure of *M. loti* MAFF303099 lipid A was elucidated as shown in Fig. 6A. It consists of two GlcNN subunits, a GalA moiety, a phosphate group, and six fatty acids including long chain ester-linked fatty acids. Accordingly, the HF-treated lipid A lacked a phosphate group, and ester-linked fatty acids were absent from the HZ-treated lipid A. These results agreed with the above MALDI-TOF-MS observations (Fig. 2).

Therefore, we investigated whether the phosphate group and ester-linked fatty acids of lipid A affect its NO inducing ability. The roots of *L. japonicus* were treated with the lipid A fractions. Three groups (23 or 24 roots in total) were stimulated, and the proportion of NO-producing roots was assessed in each group, as summarized in Fig. 6C. All of the *M. loti* lipid A fractions significantly induced NO production compared to water (mock). The proportion of the UT lipid A fraction was higher than those of the other groups in each assay; however, no significant differences in the proportions of NO producing roots were observed among the three *M. loti* lipid A fractions (UT vs HF: *p*=0.17, UT vs HZ: *p*=0.33). In contrast, the proportion of the *E. coli* lipid A fraction was significantly higher than those of the *M. loti* lipid A fractions. These data suggest that the NO-inducing activity of lipid A is not specific to *M. loti*, but requires a specific structural motif such as a phosphate group or a particular type of fatty acid.

## Discussion

The structures of several lipid A molecules from rhizobia have been elucidated previously (7). Among them, the structure of *M. loti* MAFF303099 lipid A (Fig. 6A) was found to be almost the same as that of *M. huakuii* IFO1254 (6). Its structure was also consistent with its previously reported chemical components (19). As Choma *et al.* (6) mentioned in their report, the genes of *M. loti*, including those involved in LPS biosynthesis, are very similar to those of *M. huakuii*, indicating that the above-mentioned results are reasonable. The only structural difference between the two lipid A molecules was the partial methylation of the phosphate group in the lipid A molecule of *M. loti*. Phosphate group methylation was also observed in the lipid A molecule of *Leptospira interrogans* by Que-Gewirth *et al.* (17). They further reported that the methylation was caused by the lmtA gene (4); however, no similar genes were found in the *M. loti* genome by BLAST analysis. Further gene analysis of the *M. loti* genome is thus required.

LPS from symbiotic bacteria can affect the symbiotic responses of plants (16). In LPS molecules, the lipid A moiety is one of the structures responsible for specific responses. Several studies have shown that the ester-bound long chain fatty acids in the lipid A molecule of *Rhizobium leguminosarum* modulated nodulation (2, 26). In the present study, we showed that NO production was influenced by the structure of lipid A (Fig. 6C). *E. coli* lipid A displayed the most active NO induction, and HF-treated *M. loti* lipid A had the weakest effect. The former consists of two glucosamine (GlcNN) moieties, two phosphate groups, and six ordinary length fatty acids, whereas the latter is composed of two GlcNN molecules, a GalA moiety, and six fatty acids, including long chain fatty acids (Fig. 6A, B). Although no statistically significant difference in the NO-inducing activities of the various *M. loti* lipid A fractions was observed, probably due to individual differences in the experiments, anionic groups such as phosphate groups might be responsible for the NO-inducing activity of lipid A. In addition, ester-linked fatty acids might modify the NO-inducing activity of lipid A.

In the mammalian system, lipid A is recognized by many receptors including lipopolysaccharide-binding protein (20), CD-14 (24), and Toll-like receptor-4 in combination with the adapter molecule MD-2 (21). Moreover, the structure of lipid A has been shown to affect the response of the mammalian immune system. Hajjar *et al.* (9) reported that MD-2 is able to discriminate between lipid A acylation patterns. Since lipopolysaccharide-binding proteins consist of a cationic LPS binding domain and an apolar lipid-binding pocket (20), their binding affinities might be influenced by the structure of lipid A. Although no receptor for LPS has been identified in plants, such a recognition system must control plant responses to LPS.

In conclusion, we elucidated the structure of NO-inducing *M. loti* lipid A and showed that *L. japonicus* appeared to recognize its structure. Since lipid A continuously induces NO expression (13), the lipid A moiety itself could be a pathogenic factor in the free-living form of *M. loti*; therefore, an unknown mechanism that masks the NO-inducing activity of the nodulation-inducing form of lipid A must exist in bacteria or plants. A structural modification, such as methylation on the phosphate group, might be a possible explanation for the mechanism. To understand the function of lipid A in *Rhizobium*–legume symbiosis, it will be necessary to identify the mechanisms involved in lipid A recognition in plants.

## Figures and Tables

**Table 1:** ^1^H and ^13^C-NMR chemical shifts and coupling constants of the sugar backbones of *M. loti* lipid A. Spectra were recorded at 600 MHz (^1^H) and 151 MHz (^13^C) in DMSO-d_6_/CDCl_3_ (1:1 [v/v]) at 323K

Residue positions	β-GlcNN^a^ (C)		α-GlcNN (B)		α-GalA (A)	
		
	^1^H	^13^C	^1^H	^13^C	^1^H	^13^C
Untreated lipid A						
1	4.46 (8.2)	102.9 (161.6)	4.86 (3.8)	92.9 (177.1)	4.98 (3.5)	95.4 (176.3)
2	3.63	54.9	3.84	52.4	3.76	68.6
NH	7.41		7.43			
3	4.00	54.3	4.10	52.1	3.92	69.3
NH	7.35		7.39			
4	4.06	72.0	3.29	69.3	4.08	71.2
5	3.26	77.6	3.97	72.5	4.39	71.6
6	3.64	62.2	3.60	69.1	—	171.2
	3.71	—	3.85	—		
POMe	3.44 (10.5)	53.4				
HF-treated lipid A						
1	4.38 (8.1)	102.7	4.86 (3.2)	93.0	4.98 (3.6)	95.3
2	3.61	54.1	3.85	52.4	3.76	68.5
NH	7.41		7.43			
3	3.74	56.8	4.10	52.1	3.92	(69.4)
NH	7.48		7.40			
4	3.25	69.8	3.30	69.3	4.09	71.2
5	3.16	78.7	3.97	72.5	4.41	71.5
6	3.52	61.9	3.60	69.0	—	ND^b^
	3.68	—	3.88	—		
HZ-treated lipid A						
1	4.42 (8.2)	103.2	4.85 (3.4)	92.9	4.98 (3.3)	95.1
2	3.76	54.5	3.83	52.4	3.75	68.4
NH	7.41		7.44			
3	4.01	54.6	4.08	51.8	3.92	(69.3)
NH	7.35		7.39			
4	4.08	72.1	3.44	68.3	4.09	71.1
5	3.29	77.4	3.94	72.1	4.40	71.5
6	3.65	61.2	3.59	69.3	—	171.1
	3.68	—	3.90	—		
POMe	3.51 (10.9)	53.9				

a GlcNN: 2,3-diamino-2,3-dideoxyglucose (presumed)

b ND: not determined
